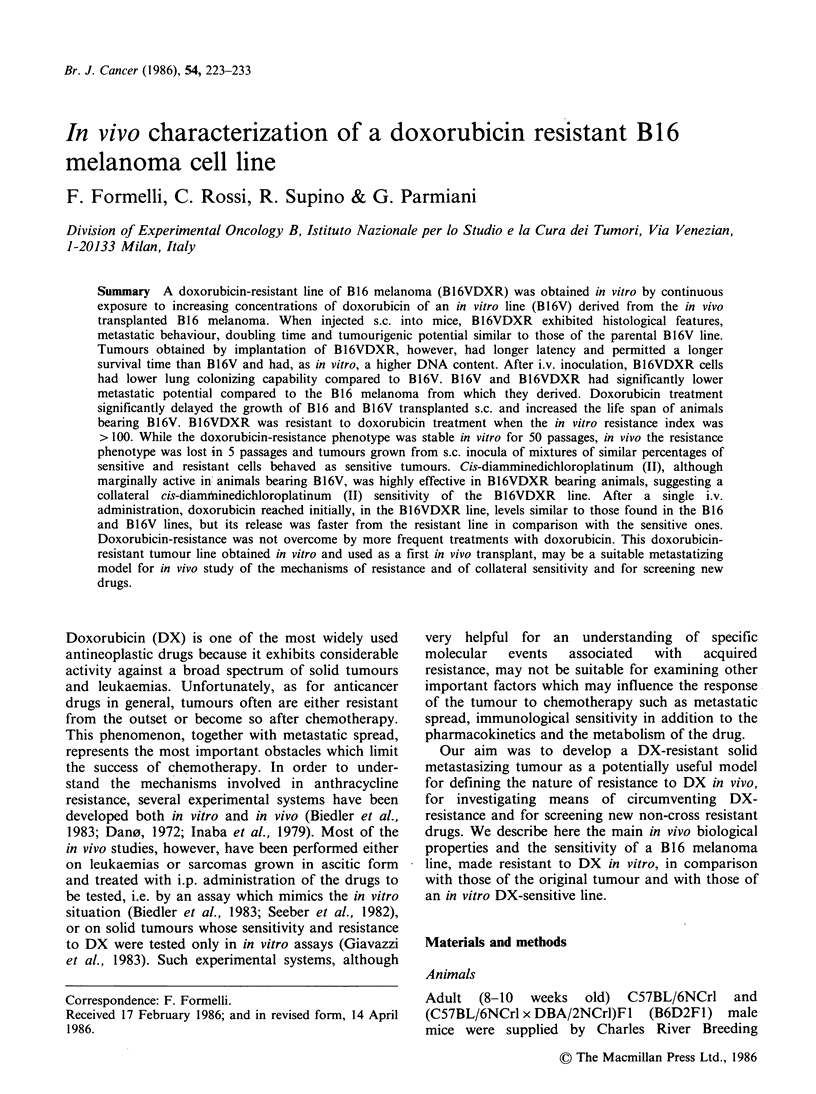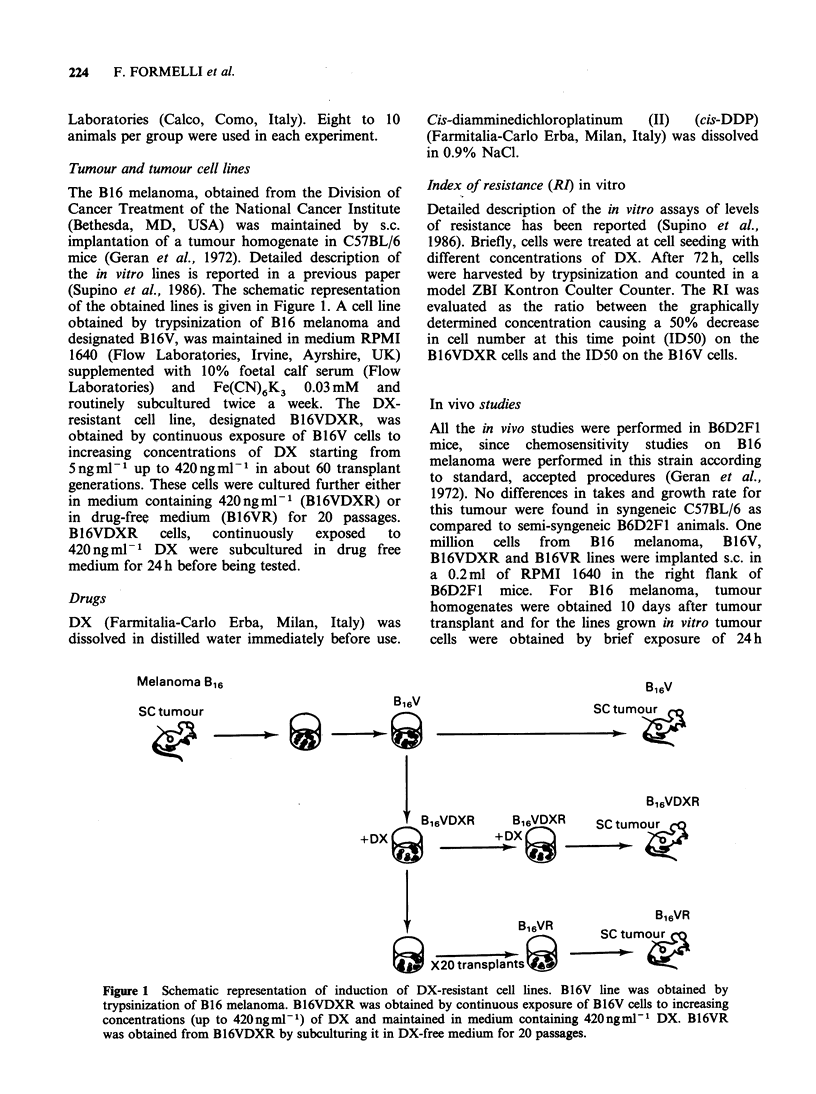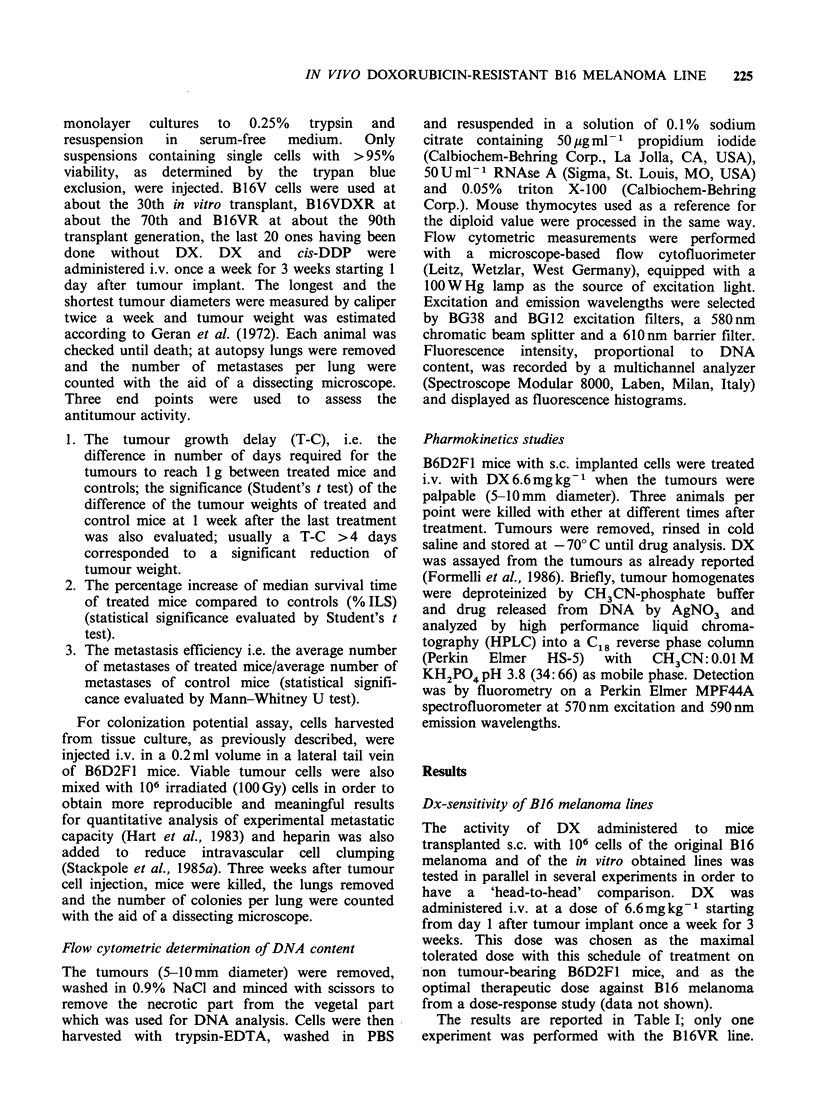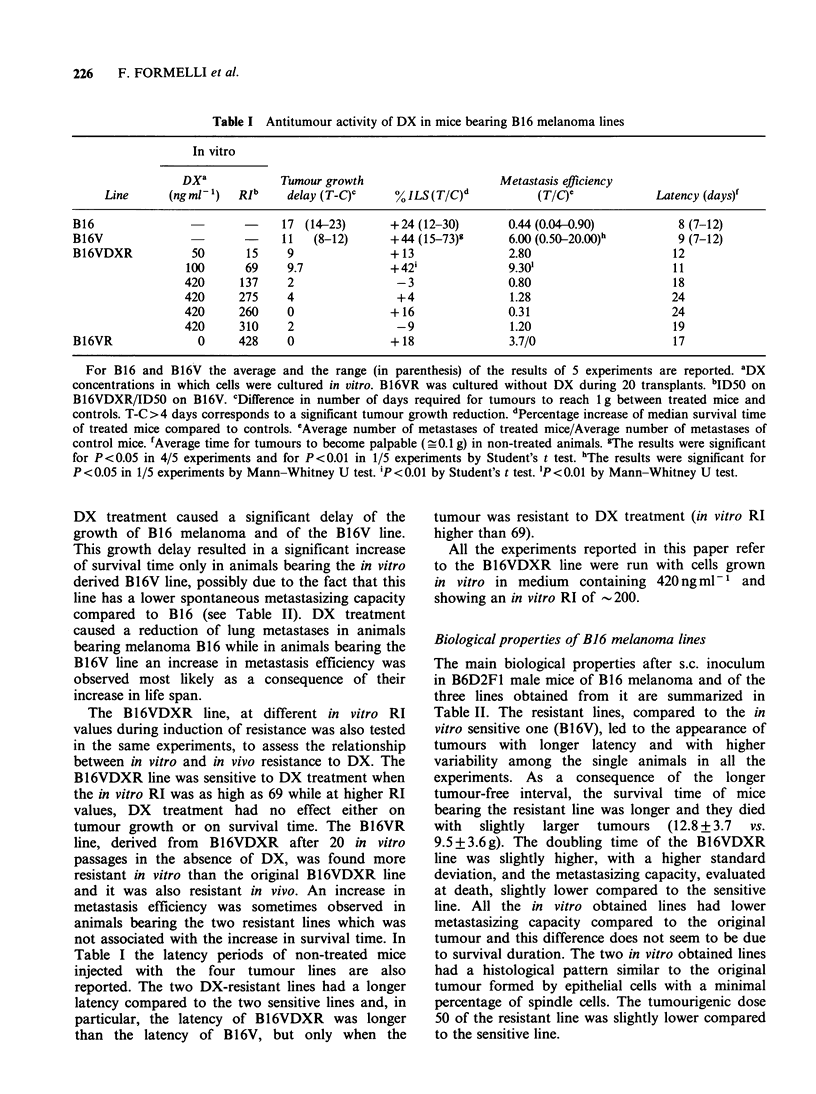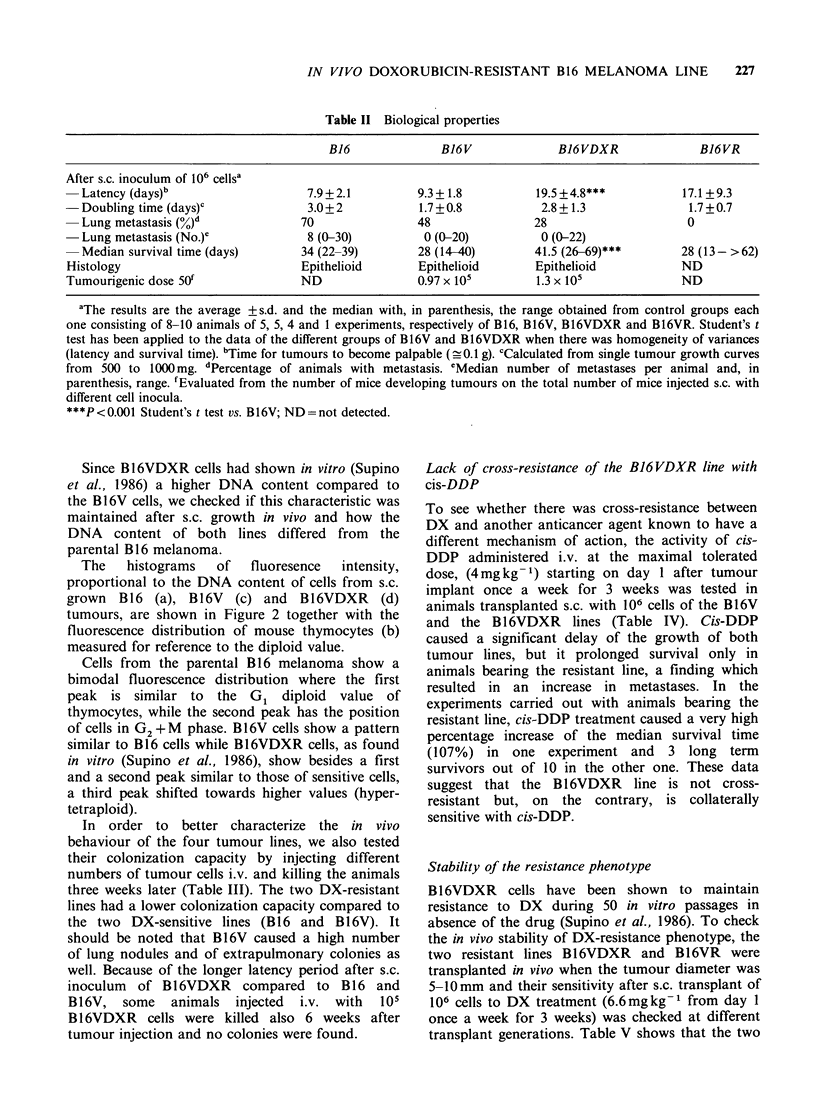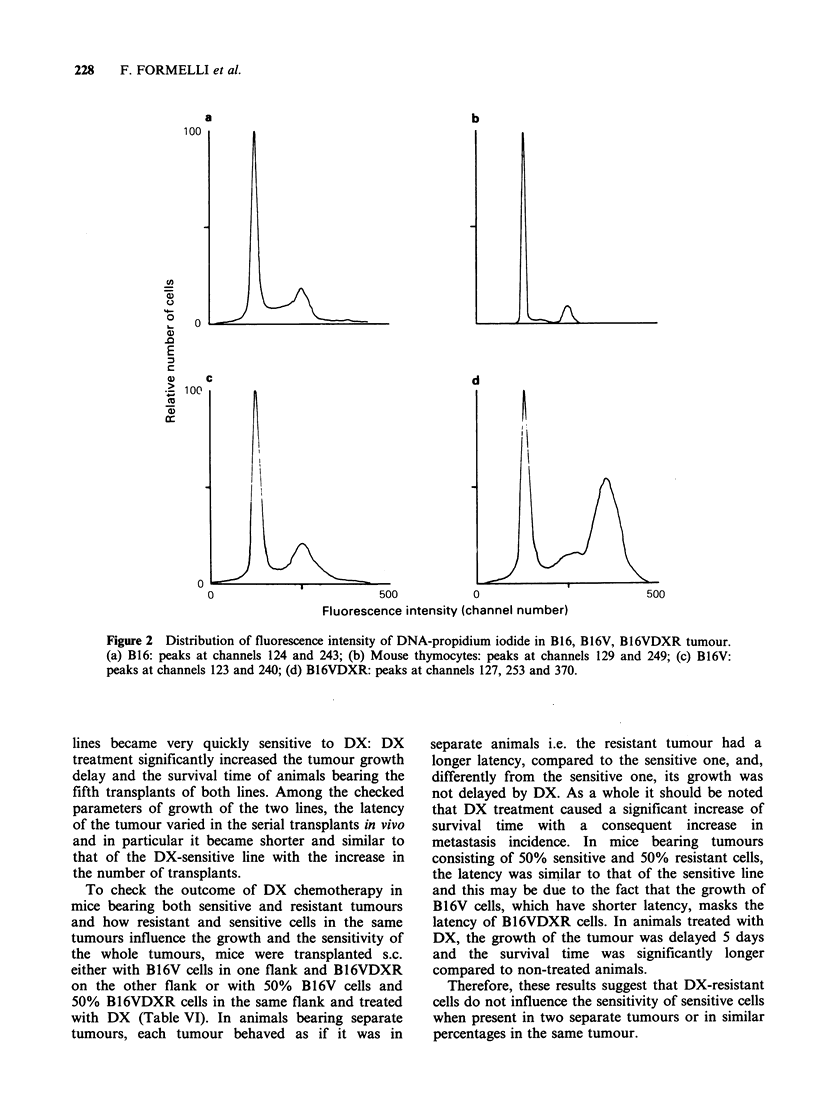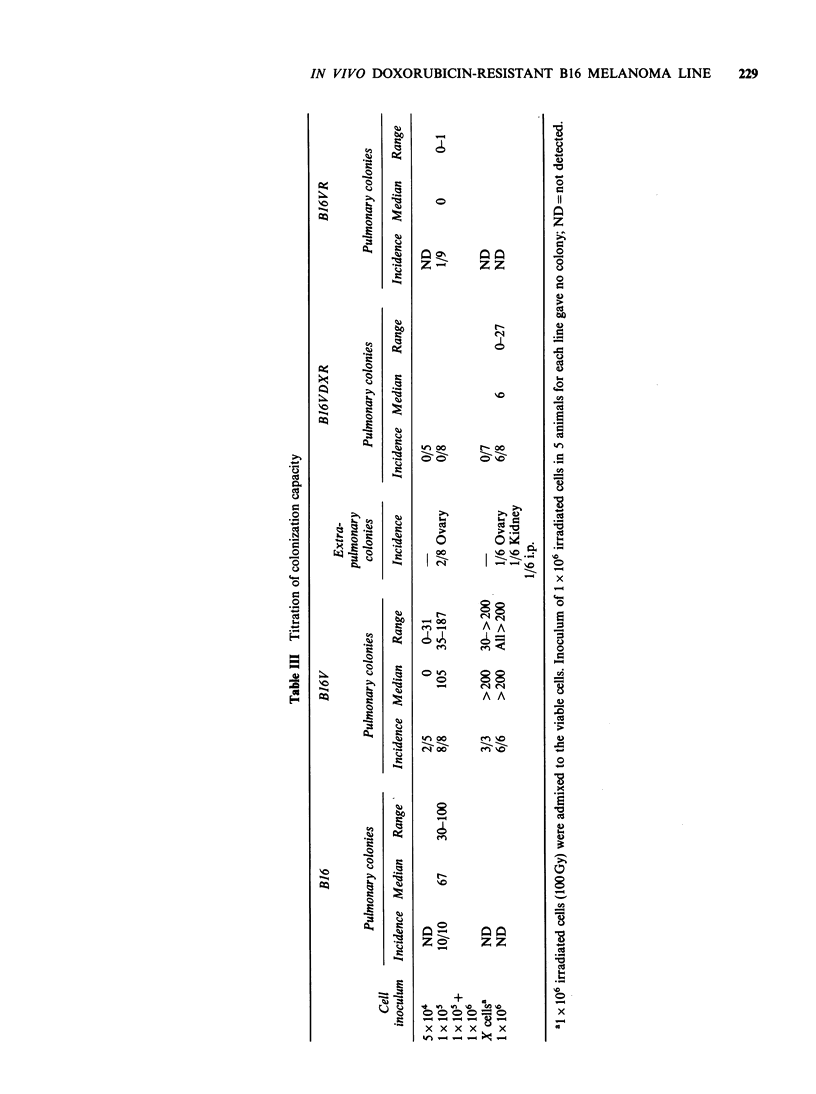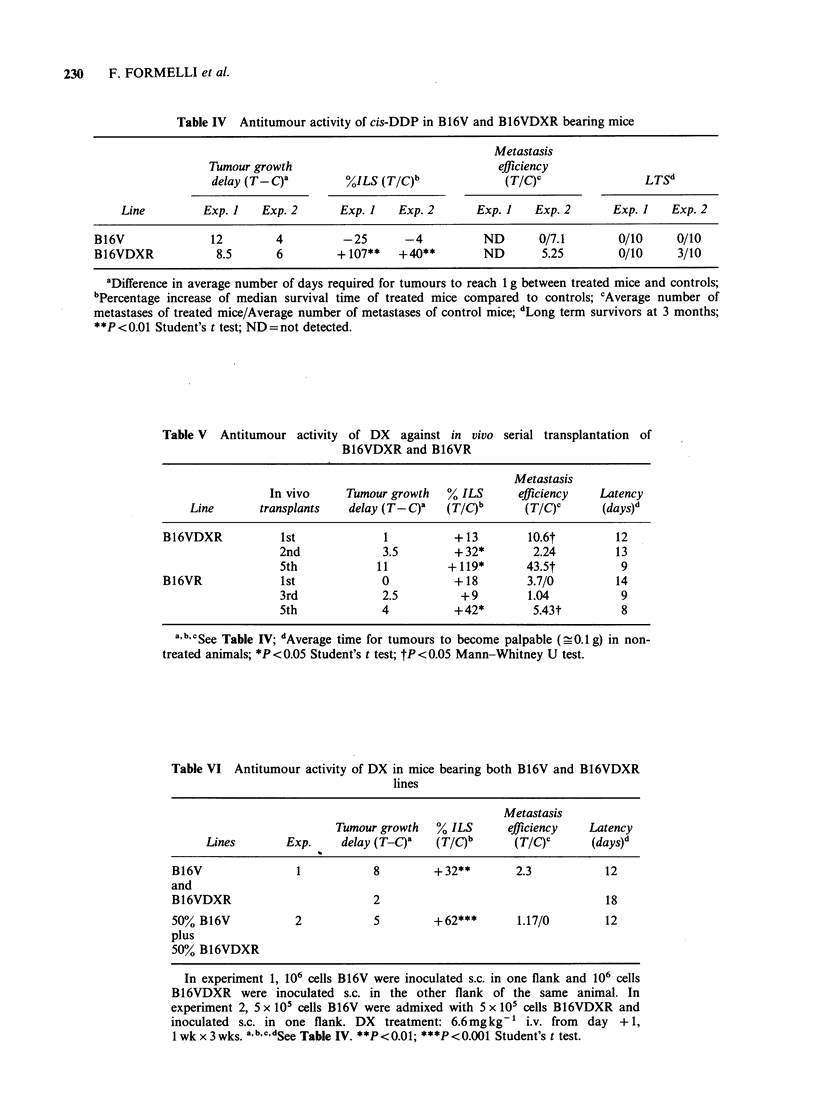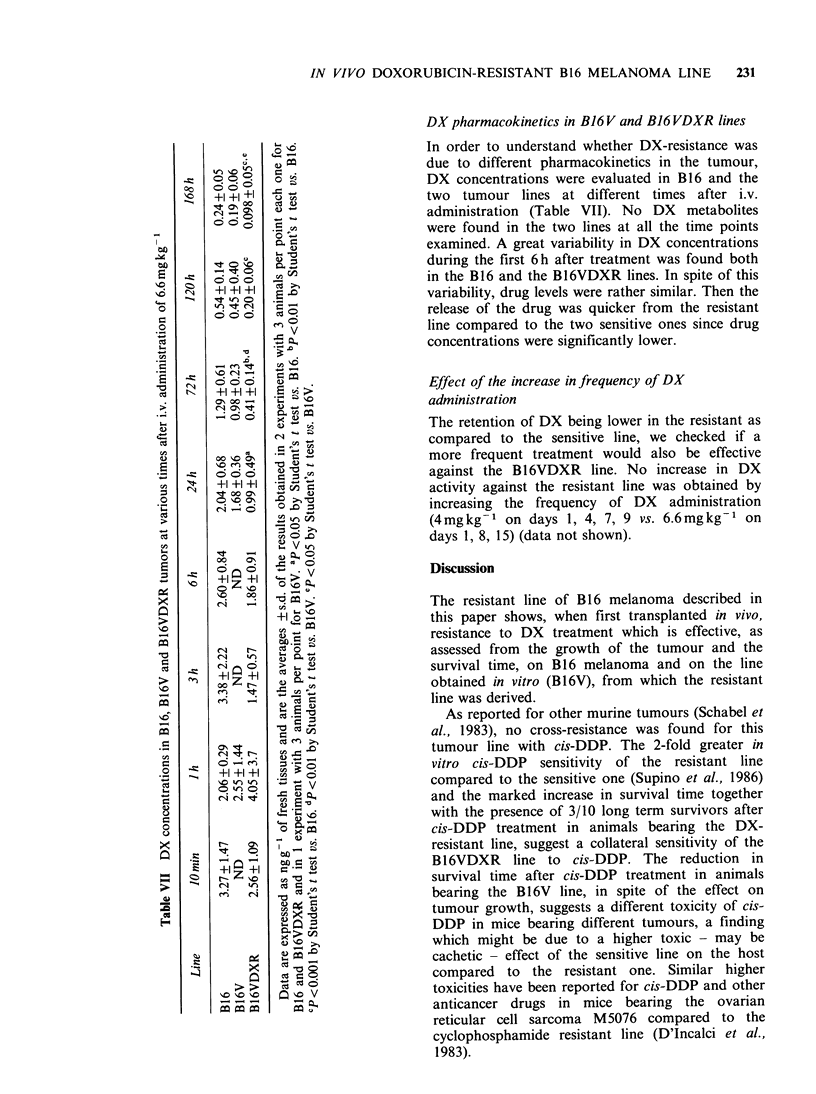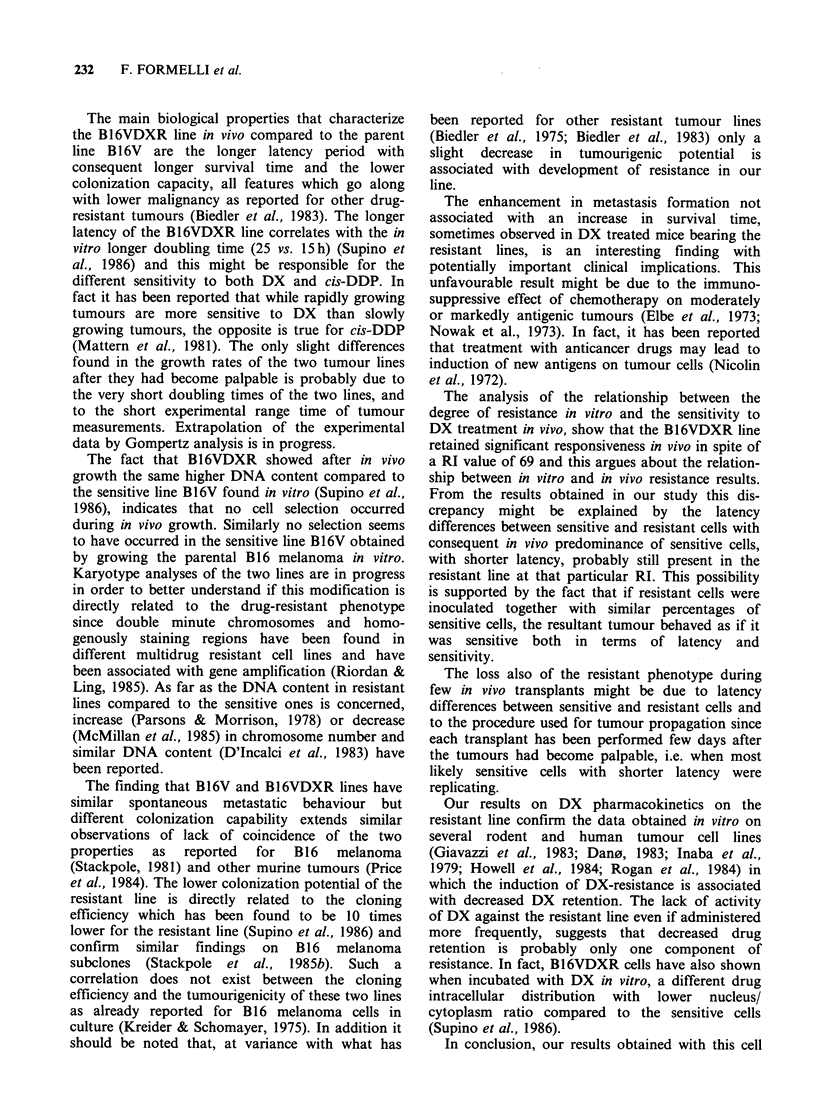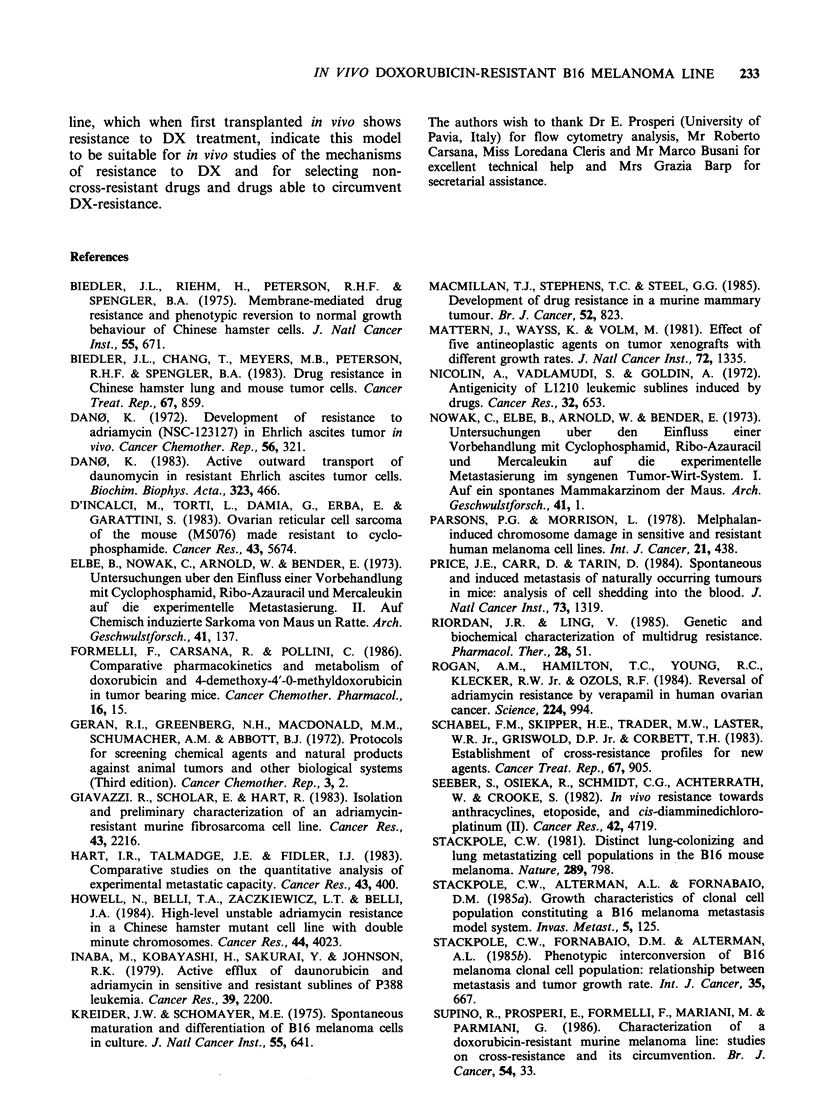# In vivo characterization of a doxorubicin resistant B16 melanoma cell line.

**DOI:** 10.1038/bjc.1986.166

**Published:** 1986-08

**Authors:** F. Formelli, C. Rossi, R. Supino, G. Parmiani

## Abstract

A doxorubicin-resistant line of B16 melanoma (B16VDXR) was obtained in vitro by continuous exposure to increasing concentrations of doxorubicin of an in vitro line (B16V) derived from the in vivo transplanted B16 melanoma. When injected s.c. into mice, B16VDXR exhibited histological features, metastatic behaviour, doubling time and tumourigenic potential similar to those of the parental B16V line. Tumours obtained by implantation of B16VDXR, however, had longer latency and permitted a longer survival time than B16V and had, as in vitro, a higher DNA content. After i.v. inoculation, B16VDXR cells had lower lung colonizing capability compared to B16V. B16V and B16VDXR had significantly lower metastatic potential compared to the B16 melanoma from which they derived. Doxorubicin treatment significantly delayed the growth of B16 and B16V transplanted s.c. and increased the life span of animals bearing B16V. B16VDXR was resistant to doxorubicin treatment when the in vitro resistance index was greater than 100. While the doxorubicin-resistance phenotype was stable in vitro for 50 passages, in vivo the resistance phenotype was lost in 5 passages and tumours grown from s.c. inocula of mixtures of similar percentages of sensitive and resistant cells behaved as sensitive tumours. Cis-diamminedichloroplatinum (II), although marginally active in animals bearing B16V, was highly effective in B16VDXR bearing animals, suggesting a collateral cis-diamminedichloroplatinum (II) sensitivity of the B16VDXR line. After a single i.v. administration, doxorubicin reached initially, in the B16VDXR line, levels similar to those found in the B16 and B16V lines, but its release was faster from the resistant line in comparison with the sensitive ones. Doxorubicin-resistance was not overcome by more frequent treatments with doxorubicin. This doxorubicin-resistant tumour line obtained in vitro and used as a first in vivo transplant, may be a suitable metastaizing model for in vivo study of the mechanisms of resistance and of collateral sensitivity and for screening new drugs.


*Br. J. Cancer* (1986), **54**, 223–233

### *In vivo* characterization of a doxorubicin resistant B16 melanoma cell line

F. Formelli, C. Rossi, R. Supino & G. Parmiani

*Division of Experimental Oncology B, Istituto Nazionale per lo Studio e la Cura dei Tumori, Via Venezian, 1-20133 Milan, Italy*

**Summary** A doxorubicin-resistant line of B16 melanoma (B16VDXR) was obtained *in vitro* by continuous exposure to increasing concentrations of doxorubicin of an *in vitro* line (B16V) derived from the *in vivo* transplanted B16 melanoma. When injected s.c. into mice, B16VDXR exhibited histological features, metastatic behaviour, doubling time and tumourigenic potential similar to those of the parental B16V line. Tumours obtained by implantation of B16VDXR, however, had longer latency and permitted a longer survival time than B16V and had, as *in vitro*, a higher DNA content. After i.v. inoculation, B16VDXR cells had lower lung colonizing capability compared to B16V. B16V and B16VDXR had significantly lower metastatic potential compared to the B16 melanoma from which they derived. Doxorubicin treatment significantly delayed the growth of B16 and B16V transplanted s.c. and increased the life span of animals bearing B16V. B16VDXR was resistant to doxorubicin treatment when the *in vitro* resistance index was >100. While the doxorubicin-resistance phenotype was stable *in vitro* for 50 passages, *in vivo* the resistance phenotype was lost in 5 passages and tumours grown from s.c. inocula of mixtures of similar percentages of sensitive and resistant cells behaved as sensitive tumours. *Cis*-diamminedichloroplatinum (II), although marginally active in animals bearing B16V, was highly effective in B16VDXR bearing animals, suggesting a collateral *cis*-diamminedichloroplatinum (II) sensitivity of the B16VDXR line. After a single i.v. administration, doxorubicin reached initially, in the B16VDXR line, levels similar to those found in the B16 and B16V lines, but its release was faster from the resistant line in comparison with the sensitive ones. Doxorubicin-resistance was not overcome by more frequent treatments with doxorubicin. This doxorubicin-resistant tumour line obtained *in vitro* and used as a first *in vivo* transplant, may be a suitable metastatizing model for *in vivo* study of the mechanisms of resistance and of collateral sensitivity and for screening new drugs.

Doxorubicin (DX) is one of the most widely used antineoplastic drugs because it exhibits considerable activity against a broad spectrum of solid tumours and leukaemias. Unfortunately, as for anticancer drugs in general, tumours often are either resistant from the outset or become so after chemotherapy. This phenomenon, together with metastatic spread, represents the most important obstacles which limit the success of chemotherapy. In order to understand the mechanisms involved in anthracycline resistance, several experimental systems have been developed both *in vitro* and *in vivo* (Biedler *et al.*, 1983; Danø, 1972; Inaba *et al.*, 1979). Most of the *in vivo* studies, however, have been performed either on leukaemias or sarcomas grown in ascitic form and treated with i.p. administration of the drugs to be tested, i.e. by an assay which mimics the *in vitro* situation (Biedler *et al.*, 1983; Seeber *et al.*, 1982), or on solid tumours whose sensitivity and resistance to DX were tested only in *in vitro* assays (Giavazzi *et al.*, 1983). Such experimental systems, although very helpful for an understanding of specific molecular events associated with acquired resistance, may not be suitable for examining other important factors which may influence the response of the tumour to chemotherapy such as metastatic spread, immunological sensitivity in addition to the pharmacokinetics and the metabolism of the drug.

Our aim was to develop a DX-resistant solid metastasizing tumour as a potentially useful model for defining the nature of resistance to DX *in vivo*, for investigating means of circumventing DX-resistance and for screening new non-cross resistant drugs. We describe here the main *in vivo* biological properties and the sensitivity of a B16 melanoma line, made resistant to DX *in vitro*, in comparison with those of the original tumour and with those of an *in vitro* DX-sensitive line.

Correspondence: F. Formelli.

Received 17 February 1986; and in revised form, 14 April 1986.

### Materials and methods

### *Animals*

Adult (8–10 weeks old) C57BL/6NCrl and (C57BL/6NCrl × DBA/2NCrl)F1 (B6D2F1) male mice were supplied by Charles River Breeding

© The Macmillan Press Ltd., 1986

Laboratories (Calco, Como, Italy). Eight to 10 animals per group were used in each experiment.

*Tumour and tumour cell lines*

The B16 melanoma, obtained from the Division of Cancer Treatment of the National Cancer Institute (Bethesda, MD, USA) was maintained by s.c. implantation of a tumour homogenate in C57BL/6 mice (Geran *et al.,* 1972). Detailed description of the *in vitro* lines is reported in a previous paper (Supino *et al.,* 1986). The schematic representation of the obtained lines is given in Figure 1. A cell line obtained by trypsinization of B16 melanoma and designated B16V, was maintained in medium RPMI 1640 (Flow Laboratories, Irvine, Ayrshire, UK) supplemented with 10% foetal calf serum (Flow Laboratories) and $Fe(CN)_6K_3$ 0.03 mM and routinely subcultured twice a week. The DX-resistant cell line, designated B16VDXR, was obtained by continuous exposure of B16V cells to increasing concentrations of DX starting from 5 ng ml$^{-1}$ up to 420 ng ml$^{-1}$ in about 60 transplant generations. These cells were cultured further either in medium containing 420 ng ml$^{-1}$ (B16VDXR) or in drug-free medium (B16VR) for 20 passages. B16VDXR cells, continuously exposed to 420 ng ml$^{-1}$ DX were subcultured in drug free medium for 24 h before being tested.

*Drugs*

DX (Farmitalia-Carlo Erba, Milan, Italy) was dissolved in distilled water immediately before use. *Cis*-diamminedichloroplatinum (II) (*cis*-DDP) (Farmitalia-Carlo Erba, Milan, Italy) was dissolved in 0.9% NaCl.

*Index of resistance (RI)* in vitro

Detailed description of the *in vitro* assays of levels of resistance has been reported (Supino *et al.,* 1986). Briefly, cells were treated at cell seeding with different concentrations of DX. After 72 h, cells were harvested by trypsinization and counted in a model ZBI Kontron Coulter Counter. The RI was evaluated as the ratio between the graphically determined concentration causing a 50% decrease in cell number at this time point (ID50) on the B16VDXR cells and the ID50 on the B16V cells.

In vivo *studies*

All the *in vivo* studies were performed in B6D2F1 mice, since chemosensitivity studies on B16 melanoma were performed in this strain according to standard, accepted procedures (Geran *et al.,* 1972). No differences in takes and growth rate for this tumour were found in syngeneic C57BL/6 as compared to semi-syngeneic B6D2F1 animals. One million cells from B16 melanoma, B16V, B16VDXR and B16VR lines were implanted s.c. in a 0.2 ml of RPMI 1640 in the right flank of B6D2F1 mice. For B16 melanoma, tumour homogenates were obtained 10 days after tumour transplant and for the lines grown *in vitro* tumour cells were obtained by brief exposure of 24 h

**Figure 1** Schematic representation of induction of DX-resistant cell lines. B16V line was obtained by trypsinization of B16 melanoma. B16VDXR was obtained by continuous exposure of B16V cells to increasing concentrations (up to 420 ng ml$^{-1}$) of DX and maintained in medium containing 420 ng ml$^{-1}$ DX. B16VR was obtained from B16VDXR by subculturing it in DX-free medium for 20 passages.

monolayer cultures to 0.25% trypsin and resuspension in serum-free medium. Only suspensions containing single cells with >95% viability, as determined by the trypan blue exclusion, were injected. B16V cells were used at about the 30th *in vitro* transplant, B16VDXR at about the 70th and B16VR at about the 90th transplant generation, the last 20 ones having been done without DX. DX and *cis*-DDP were administered i.v. once a week for 3 weeks starting 1 day after tumour implant. The longest and the shortest tumour diameters were measured by caliper twice a week and tumour weight was estimated according to Geran *et al.* (1972). Each animal was checked until death; at autopsy lungs were removed and the number of metastases per lung were counted with the aid of a dissecting microscope. Three end points were used to assess the antitumour activity.

1. The tumour growth delay (T-C), i.e. the difference in number of days required for the tumours to reach 1 g between treated mice and controls; the significance (Student's *t* test) of the difference of the tumour weights of treated and control mice at 1 week after the last treatment was also evaluated; usually a T-C >4 days corresponded to a significant reduction of tumour weight.
2. The percentage increase of median survival time of treated mice compared to controls (% ILS) (statistical significance evaluated by Student's *t* test).
3. The metastasis efficiency i.e. the average number of metastases of treated mice/average number of metastases of control mice (statistical significance evaluated by Mann–Whitney U test).

For colonization potential assay, cells harvested from tissue culture, as previously described, were injected i.v. in a 0.2 ml volume in a lateral tail vein of B6D2F1 mice. Viable tumour cells were also mixed with $10^6$ irradiated (100 Gy) cells in order to obtain more reproducible and meaningful results for quantitative analysis of experimental metastatic capacity (Hart *et al.*, 1983) and heparin was also added to reduce intravascular cell clumping (Stackpole *et al.*, 1985*a*). Three weeks after tumour cell injection, mice were killed, the lungs removed and the number of colonies per lung were counted with the aid of a dissecting microscope.

*Flow cytometric determination of DNA content*

The tumours (5–10 mm diameter) were removed, washed in 0.9% NaCl and minced with scissors to remove the necrotic part from the vegetal part which was used for DNA analysis. Cells were then harvested with trypsin-EDTA, washed in PBS and resuspended in a solution of 0.1% sodium citrate containing 50 $\mu$g ml$^{-1}$ propidium iodide (Calbiochem-Behring Corp., La Jolla, CA, USA), 50 U ml$^{-1}$ RNAse A (Sigma, St. Louis, MO, USA) and 0.05% triton X-100 (Calbiochem-Behring Corp.). Mouse thymocytes used as a reference for the diploid value were processed in the same way. Flow cytometric measurements were performed with a microscope-based flow cytofluorimeter (Leitz, Wetzlar, West Germany), equipped with a 100 W Hg lamp as the source of excitation light. Excitation and emission wavelengths were selected by BG38 and BG12 excitation filters, a 580 nm chromatic beam splitter and a 610 nm barrier filter. Fluorescence intensity, proportional to DNA content, was recorded by a multichannel analyzer (Spectroscope Modular 8000, Laben, Milan, Italy) and displayed as fluorescence histograms.

*Pharmokinetics studies*

B6D2F1 mice with s.c. implanted cells were treated i.v. with DX 6.6 mg kg$^{-1}$ when the tumours were palpable (5–10 mm diameter). Three animals per point were killed with ether at different times after treatment. Tumours were removed, rinsed in cold saline and stored at −70° C until drug analysis. DX was assayed from the tumours as already reported (Formelli *et al.*, 1986). Briefly, tumour homogenates were deproteinized by $CH_3CN$-phosphate buffer and drug released from DNA by $AgNO_3$ and analyzed by high performance liquid chromatography (HPLC) into a $C_{18}$ reverse phase column (Perkin Elmer HS-5) with $CH_3CN$:0.01 M $KH_2PO_4$ pH 3.8 (34:66) as mobile phase. Detection was by fluorometry on a Perkin Elmer MPF44A spectrofluorometer at 570 nm excitation and 590 nm emission wavelengths.

### Results

*Dx-sensitivity of B16 melanoma lines*

The activity of DX administered to mice transplanted s.c. with $10^6$ cells of the original B16 melanoma and of the *in vitro* obtained lines was tested in parallel in several experiments in order to have a 'head-to-head' comparison. DX was administered i.v. at a dose of 6.6 mg kg$^{-1}$ starting from day 1 after tumour implant once a week for 3 weeks. This dose was chosen as the maximal tolerated dose with this schedule of treatment on non tumour-bearing B6D2F1 mice, and as the optimal therapeutic dose against B16 melanoma from a dose-response study (data not shown).

The results are reported in Table I; only one experiment was performed with the B16VR line.

**Table I** Antitumour activity of DX in mice bearing B16 melanoma lines

| | In vitro | | | | | |
|---|---|---|---|---|---|---|
| *Line* | *DX*[a] (*ng ml*$^{-1}$) | *RI*[b] | *Tumour growth delay* (*T-C*)[c] | %*ILS*(*T/C*)[d] | *Metastasis efficiency* (*T/C*)[e] | *Latency* (*days*)[f] |
| B16 | — | — | 17 (14–23) | +24 (12–30) | 0.44 (0.04–0.90) | 8 (7–12) |
| B16V | — | — | 11 (8–12) | +44 (15–73)[g] | 6.00 (0.50–20.00)[h] | 9 (7–12) |
| B16VDXR | 50 | 15 | 9 | +13 | 2.80 | 12 |
| | 100 | 69 | 9.7 | +42[i] | 9.30[l] | 11 |
| | 420 | 137 | 2 | −3 | 0.80 | 18 |
| | 420 | 275 | 4 | +4 | 1.28 | 24 |
| | 420 | 260 | 0 | +16 | 0.31 | 24 |
| | 420 | 310 | 2 | −9 | 1.20 | 19 |
| B16VR | 0 | 428 | 0 | +18 | 3.7/0 | 17 |

For B16 and B16V the average and the range (in parenthesis) of the results of 5 experiments are reported. [a]DX concentrations in which cells were cultured *in vitro*. B16VR was cultured without DX during 20 transplants. [b]ID50 on B16VDXR/ID50 on B16V. [c]Difference in number of days required for tumours to reach 1 g between treated mice and controls. T-C > 4 days corresponds to a significant tumour growth reduction. [d]Percentage increase of median survival time of treated mice compared to controls. [e]Average number of metastases of treated mice/Average number of metastases of control mice. [f]Average time for tumours to become palpable ($\cong$0.1 g) in non-treated animals. [g]The results were significant for $P<0.05$ in 4/5 experiments and for $P<0.01$ in 1/5 experiments by Student's *t* test. [h]The results were significant for $P<0.05$ in 1/5 experiments by Mann–Whitney U test. [i]$P<0.01$ by Student's *t* test. [l]$P<0.01$ by Mann–Whitney U test.

DX treatment caused a significant delay of the growth of B16 melanoma and of the B16V line. This growth delay resulted in a significant increase of survival time only in animals bearing the *in vitro* derived B16V line, possibly due to the fact that this line has a lower spontaneous metastasizing capacity compared to B16 (see Table II). DX treatment caused a reduction of lung metastases in animals bearing melanoma B16 while in animals bearing the B16V line an increase in metastasis efficiency was observed most likely as a consequence of their increase in life span.

The B16VDXR line, at different *in vitro* RI values during induction of resistance was also tested in the same experiments, to assess the relationship between *in vitro* and *in vivo* resistance to DX. The B16VDXR line was sensitive to DX treatment when the *in vitro* RI was as high as 69 while at higher RI values, DX treatment had no effect either on tumour growth or on survival time. The B16VR line, derived from B16VDXR after 20 *in vitro* passages in the absence of DX, was found more resistant *in vitro* than the original B16VDXR line and it was also resistant *in vivo*. An increase in metastasis efficiency was sometimes observed in animals bearing the two resistant lines which was not associated with the increase in survival time. In Table I the latency periods of non-treated mice injected with the four tumour lines are also reported. The two DX-resistant lines had a longer latency compared to the two sensitive lines and, in particular, the latency of B16VDXR was longer than the latency of B16V, but only when the tumour was resistant to DX treatment (*in vitro* RI higher than 69).

All the experiments reported in this paper refer to the B16VDXR line were run with cells grown *in vitro* in medium containing 420 ng ml$^{-1}$ and showing an *in vitro* RI of ~200.

### *Biological properties of B16 melanoma lines*

The main biological properties after s.c. inoculum in B6D2F1 male mice of B16 melanoma and of the three lines obtained from it are summarized in Table II. The resistant lines, compared to the *in vitro* sensitive one (B16V), led to the appearance of tumours with longer latency and with higher variability among the single animals in all the experiments. As a consequence of the longer tumour-free interval, the survival time of mice bearing the resistant line was longer and they died with slightly larger tumours ($12.8 \pm 3.7$ *vs.* $9.5 \pm 3.6$ g). The doubling time of the B16VDXR line was slightly higher, with a higher standard deviation, and the metastasizing capacity, evaluated at death, slightly lower compared to the sensitive line. All the *in vitro* obtained lines had lower metastasizing capacity compared to the original tumour and this difference does not seem to be due to survival duration. The two *in vitro* obtained lines had a histological pattern similar to the original tumour formed by epithelial cells with a minimal percentage of spindle cells. The tumourigenic dose 50 of the resistant line was slightly lower compared to the sensitive line.

**Table II** Biological properties

| | *B16* | *B16V* | *B16VDXR* | *B16VR* |
|---|---|---|---|---|
| After s.c. inoculum of $10^6$ cells[a] | | | | |
| — Latency (days)[b] | 7.9±2.1 | 9.3±1.8 | 19.5±4.8*** | 17.1±9.3 |
| — Doubling time (days)[c] | 3.0±2 | 1.7±0.8 | 2.8±1.3 | 1.7±0.7 |
| — Lung metastasis (%)[d] | 70 | 48 | 28 | 0 |
| — Lung metastasis (No.)[e] | 8 (0–30) | 0 (0–20) | 0 (0–22) | |
| — Median survival time (days) | 34 (22–39) | 28 (14–40) | 41.5 (26–69)*** | 28 (13– >62) |
| Histology | Epithelioid | Epithelioid | Epithelioid | ND |
| Tumourigenic dose 50[f] | ND | $0.97 \times 10^5$ | $1.3 \times 10^5$ | ND |

[a]The results are the average ±s.d. and the median with, in parenthesis, the range obtained from control groups each one consisting of 8–10 animals of 5, 5, 4 and 1 experiments, respectively of B16, B16V, B16VDXR and B16VR. Student's *t* test has been applied to the data of the different groups of B16V and B16VDXR when there was homogeneity of variances (latency and survival time). [b]Time for tumours to become palpable (≅0.1 g). [c]Calculated from single tumour growth curves from 500 to 1000 mg. [d]Percentage of animals with metastasis. [e]Median number of metastases per animal and, in parenthesis, range. [f]Evaluated from the number of mice developing tumours on the total number of mice injected s.c. with different cell inocula.
***$P<0.001$ Student's *t* test *vs.* B16V; ND = not detected.

Since B16VDXR cells had shown *in vitro* (Supino *et al.*, 1986) a higher DNA content compared to the B16V cells, we checked if this characteristic was maintained after s.c. growth *in vivo* and how the DNA content of both lines differed from the parental B16 melanoma.

The histograms of fluoresence intensity, proportional to the DNA content of cells from s.c. grown B16 (a), B16V (c) and B16VDXR (d) tumours, are shown in Figure 2 together with the fluorescence distribution of mouse thymocytes (b) measured for reference to the diploid value.

Cells from the parental B16 melanoma show a bimodal fluorescence distribution where the first peak is similar to the $G_1$ diploid value of thymocytes, while the second peak has the position of cells in $G_2+M$ phase. B16V cells show a pattern similar to B16 cells while B16VDXR cells, as found *in vitro* (Supino *et al.*, 1986), show besides a first and a second peak similar to those of sensitive cells, a third peak shifted towards higher values (hypertetraploid).

In order to better characterize the *in vivo* behaviour of the four tumour lines, we also tested their colonization capacity by injecting different numbers of tumour cells i.v. and killing the animals three weeks later (Table III). The two DX-resistant lines had a lower colonization capacity compared to the two DX-sensitive lines (B16 and B16V). It should be noted that B16V caused a high number of lung nodules and of extrapulmonary colonies as well. Because of the longer latency period after s.c. inoculum of B16VDXR compared to B16 and B16V, some animals injected i.v. with $10^5$ B16VDXR cells were killed also 6 weeks after tumour injection and no colonies were found.

### *Lack of cross-resistance of the B16VDXR line with* cis-*DDP*

To see whether there was cross-resistance between DX and another anticancer agent known to have a different mechanism of action, the activity of *cis*-DDP administered i.v. at the maximal tolerated dose, ($4\,mg\,kg^{-1}$) starting on day 1 after tumour implant once a week for 3 weeks was tested in animals transplanted s.c. with $10^6$ cells of the B16V and the B16VDXR lines (Table IV). *Cis*-DDP caused a significant delay of the growth of both tumour lines, but it prolonged survival only in animals bearing the resistant line, a finding which resulted in an increase in metastases. In the experiments carried out with animals bearing the resistant line, *cis*-DDP treatment caused a very high percentage increase of the median survival time (107%) in one experiment and 3 long term survivors out of 10 in the other one. These data suggest that the B16VDXR line is not cross-resistant but, on the contrary, is collaterally sensitive with *cis*-DDP.

### *Stability of the resistance phenotype*

B16VDXR cells have been shown to maintain resistance to DX during 50 *in vitro* passages in absence of the drug (Supino *et al.*, 1986). To check the *in vivo* stability of DX-resistance phenotype, the two resistant lines B16VDXR and B16VR were transplanted *in vivo* when the tumour diameter was 5–10 mm and their sensitivity after s.c. transplant of $10^6$ cells to DX treatment ($6.6\,mg\,kg^{-1}$ from day 1 once a week for 3 weeks) was checked at different transplant generations. Table V shows that the two

**Figure 2** Distribution of fluorescence intensity of DNA-propidium iodide in B16, B16V, B16VDXR tumour. (a) B16: peaks at channels 124 and 243; (b) Mouse thymocytes: peaks at channels 129 and 249; (c) B16V: peaks at channels 123 and 240; (d) B16VDXR: peaks at channels 127, 253 and 370.

lines became very quickly sensitive to DX: DX treatment significantly increased the tumour growth delay and the survival time of animals bearing the fifth transplants of both lines. Among the checked parameters of growth of the two lines, the latency of the tumour varied in the serial transplants *in vivo* and in particular it became shorter and similar to that of the DX-sensitive line with the increase in the number of transplants.

To check the outcome of DX chemotherapy in mice bearing both sensitive and resistant tumours and how resistant and sensitive cells in the same tumours influence the growth and the sensitivity of the whole tumours, mice were transplanted s.c. either with B16V cells in one flank and B16VDXR on the other flank or with 50% B16V cells and 50% B16VDXR cells in the same flank and treated with DX (Table VI). In animals bearing separate tumours, each tumour behaved as if it was in separate animals i.e. the resistant tumour had a longer latency, compared to the sensitive one, and, differently from the sensitive one, its growth was not delayed by DX. As a whole it should be noted that DX treatment caused a significant increase of survival time with a consequent increase in metastasis incidence. In mice bearing tumours consisting of 50% sensitive and 50% resistant cells, the latency was similar to that of the sensitive line and this may be due to the fact that the growth of B16V cells, which have shorter latency, masks the latency of B16VDXR cells. In animals treated with DX, the growth of the tumour was delayed 5 days and the survival time was significantly longer compared to non-treated animals.

Therefore, these results suggest that DX-resistant cells do not influence the sensitivity of sensitive cells when present in two separate tumours or in similar percentages in the same tumour.

**Table III** Titration of colonization capacity

| Cell inoculum | *B16* Pulmonary colonies | | | *B16V* Pulmonary colonies | | | *B16V* Extra-pulmonary colonies | *B16VDXR* Pulmonary colonies | | | *B16VR* Pulmonary colonies | | |
|---|---|---|---|---|---|---|---|---|---|---|---|---|---|
| | *Incidence* | *Median* | *Range* | *Incidence* | *Median* | *Range* | *Incidence* | *Incidence* | *Median* | *Range* | *Incidence* | *Median* | *Range* |
| $5\times10^4$ | ND | | | 2/5 | 0 | 0–31 | — | 0/5 | | | ND | | |
| $1\times10^5$ | 10/10 | 67 | 30–100 | 8/8 | 105 | 35–187 | 2/8 Ovary | 0/8 | | | 1/9 | 0 | 0–1 |
| $1\times10^5$ + $1\times10^6$ *X* cells[a] | ND | | | 3/3 | >200 | 30–>200 | — | 0/7 | | | ND | | |
| $1\times10^6$ | ND | | | 6/6 | >200 | All>200 | 1/6 Ovary<br>1/6 Kidney<br>1/6 i.p. | 6/8 | 6 | 0–27 | ND | | |

[a] $1\times10^6$ irradiated cells (100 Gy) were admixed to the viable cells. Inoculum of $1\times10^6$ irradiated cells in 5 animals for each line gave no colony; ND = not detected.

**Table IV** Antitumour activity of *cis*-DDP in B16V and B16VDXR bearing mice

| *Line* | *Tumour growth delay (T − C)*[a] | | *%ILS (T/C)*[b] | | *Metastasis efficiency (T/C)*[c] | | *LTS*[d] | |
|---|---|---|---|---|---|---|---|---|
| | *Exp. 1* | *Exp. 2* | *Exp. 1* | *Exp. 2* | *Exp. 1* | *Exp. 2* | *Exp. 1* | *Exp. 2* |
| B16V | 12 | 4 | −25 | −4 | ND | 0/7.1 | 0/10 | 0/10 |
| B16VDXR | 8.5 | 6 | +107** | +40** | ND | 5.25 | 0/10 | 3/10 |

[a]Difference in average number of days required for tumours to reach 1 g between treated mice and controls; [b]Percentage increase of median survival time of treated mice compared to controls; [c]Average number of metastases of treated mice/Average number of metastases of control mice; [d]Long term survivors at 3 months; $^{**}P<0.01$ Student's $t$ test; ND = not detected.

**Table V** Antitumour activity of DX against *in vivo* serial transplantation of B16VDXR and B16VR

| *Line* | In vivo *transplants* | *Tumour growth delay (T − C)*[a] | *% ILS (T/C)*[b] | *Metastasis efficiency (T/C)*[c] | *Latency (days)*[d] |
|---|---|---|---|---|---|
| B16VDXR | 1st | 1 | +13 | 10.6† | 12 |
| | 2nd | 3.5 | +32* | 2.24 | 13 |
| | 5th | 11 | +119* | 43.5† | 9 |
| B16VR | 1st | 0 | +18 | 3.7/0 | 14 |
| | 3rd | 2.5 | +9 | 1.04 | 9 |
| | 5th | 4 | +42* | 5.43† | 8 |

[a,b,c]See **Table IV**; [d]Average time for tumours to become palpable ($\cong 0.1$ g) in non-treated animals; $^{*}P<0.05$ Student's $t$ test; $^{\dagger}P<0.05$ Mann–Whitney U test.

**Table VI** Antitumour activity of DX in mice bearing both B16V and B16VDXR lines

| *Lines* | *Exp.* | *Tumour growth delay (T–C)*[a] | *% ILS (T/C)*[b] | *Metastasis efficiency (T/C)*[c] | *Latency (days)*[d] |
|---|---|---|---|---|---|
| B16V | 1 | 8 | +32** | 2.3 | 12 |
| and | | | | | |
| B16VDXR | | 2 | | | 18 |
| 50% B16V | 2 | 5 | +62*** | 1.17/0 | 12 |
| plus | | | | | |
| 50% B16VDXR | | | | | |

In experiment 1, $10^6$ cells B16V were inoculated s.c. in one flank and $10^6$ cells B16VDXR were inoculated s.c. in the other flank of the same animal. In experiment 2, $5\times10^5$ cells B16V were admixed with $5\times10^5$ cells B16VDXR and inoculated s.c. in one flank. DX treatment: 6.6 mg kg$^{-1}$ i.v. from day +1, 1 wk × 3 wks. [a,b,c,d]See **Table IV**. $^{**}P<0.01$; $^{***}P<0.001$ Student's $t$ test.

**Table VII** DX concentrations in B16, B16V and B16VDXR tumors at various times after i.v. administration of $6.6\,mg\,kg^{-1}$

| *Line* | *10 min* | *1 h* | *3 h* | *6 h* | *24 h* | *72 h* | *120 h* | *168 h* |
|---|---|---|---|---|---|---|---|---|
| B16 | $3.27 \pm 1.47$ | $2.06 \pm 0.29$ | $3.38 \pm 2.22$ | $2.60 \pm 0.84$ | $2.04 \pm 0.68$ | $1.29 \pm 0.61$ | $0.54 \pm 0.14$ | $0.24 \pm 0.05$ |
| B16V | ND | $2.55 \pm 1.44$ | ND | ND | $1.68 \pm 0.36$ | $0.98 \pm 0.23$ | $0.45 \pm 0.40$ | $0.19 \pm 0.06$ |
| B16VDXR | $2.56 \pm 1.09$ | $4.05 \pm 3.7$ | $1.47 \pm 0.57$ | $1.86 \pm 0.91$ | $0.99 \pm 0.49^{a}$ | $0.41 \pm 0.14^{b,d}$ | $0.20 \pm 0.06^{c}$ | $0.098 \pm 0.05^{c,e}$ |

Data are expressed as $ng\,g^{-1}$ of fresh tissues and are the averages ±s.d. of the results obtained in 2 experiments with 3 animals per point each one for B16 and B16VDXR and in 1 experiment with 3 animals per point for B16V. $^{a}P<0.05$ by Student's *t* test *vs.* B16. $^{b}P<0.01$ by Student's *t* test *vs.* B16. $^{c}P<0.001$ by Student's *t* test *vs.* B16. $^{d}P<0.01$ by Student's *t* test *vs.* B16V. $^{e}P<0.05$ by Student's *t* test *vs.* B16V.

*DX pharmacokinetics in B16V and B16VDXR lines*

In order to understand whether DX-resistance was due to different pharmacokinetics in the tumour, DX concentrations were evaluated in B16 and the two tumour lines at different times after i.v. administration (Table VII). No DX metabolites were found in the two lines at all the time points examined. A great variability in DX concentrations during the first 6 h after treatment was found both in the B16 and the B16VDXR lines. In spite of this variability, drug levels were rather similar. Then the release of the drug was quicker from the resistant line compared to the two sensitive ones since drug concentrations were significantly lower.

*Effect of the increase in frequency of DX administration*

The retention of DX being lower in the resistant as compared to the sensitive line, we checked if a more frequent treatment would also be effective against the B16VDXR line. No increase in DX activity against the resistant line was obtained by increasing the frequency of DX administration ($4\,mg\,kg^{-1}$ on days 1, 4, 7, 9 *vs.* $6.6\,mg\,kg^{-1}$ on days 1, 8, 15) (data not shown).

### Discussion

The resistant line of B16 melanoma described in this paper shows, when first transplanted *in vivo*, resistance to DX treatment which is effective, as assessed from the growth of the tumour and the survival time, on B16 melanoma and on the line obtained *in vitro* (B16V), from which the resistant line was derived.

As reported for other murine tumours (Schabel *et al.*, 1983), no cross-resistance was found for this tumour line with *cis*-DDP. The 2-fold greater *in vitro cis*-DDP sensitivity of the resistant line compared to the sensitive one (Supino *et al.*, 1986) and the marked increase in survival time together with the presence of 3/10 long term survivors after *cis*-DDP treatment in animals bearing the DX-resistant line, suggest a collateral sensitivity of the B16VDXR line to *cis*-DDP. The reduction in survival time after *cis*-DDP treatment in animals bearing the B16V line, in spite of the effect on tumour growth, suggests a different toxicity of *cis*-DDP in mice bearing different tumours, a finding which might be due to a higher toxic – may be cachetic – effect of the sensitive line on the host compared to the resistant one. Similar higher toxicities have been reported for *cis*-DDP and other anticancer drugs in mice bearing the ovarian reticular cell sarcoma M5076 compared to the cyclophosphamide resistant line (D'Incalci *et al.*, 1983).

The main biological properties that characterize the B16VDXR line *in vivo* compared to the parent line B16V are the longer latency period with consequent longer survival time and the lower colonization capacity, all features which go along with lower malignancy as reported for other drug-resistant tumours (Biedler *et al.*, 1983). The longer latency of the B16VDXR line correlates with the *in vitro* longer doubling time (25 *vs.* 15 h) (Supino *et al.*, 1986) and this might be responsible for the different sensitivity to both DX and *cis*-DDP. In fact it has been reported that while rapidly growing tumours are more sensitive to DX than slowly growing tumours, the opposite is true for *cis*-DDP (Mattern *et al.*, 1981). The only slight differences found in the growth rates of the two tumour lines after they had become palpable is probably due to the very short doubling times of the two lines, and to the short experimental range time of tumour measurements. Extrapolation of the experimental data by Gompertz analysis is in progress.

The fact that B16VDXR showed after *in vivo* growth the same higher DNA content compared to the sensitive line B16V found *in vitro* (Supino *et al.*, 1986), indicates that no cell selection occurred during *in vivo* growth. Similarly no selection seems to have occurred in the sensitive line B16V obtained by growing the parental B16 melanoma *in vitro*. Karyotype analyses of the two lines are in progress in order to better understand if this modification is directly related to the drug-resistant phenotype since double minute chromosomes and homogenously staining regions have been found in different multidrug resistant cell lines and have been associated with gene amplification (Riordan & Ling, 1985). As far as the DNA content in resistant lines compared to the sensitive ones is concerned, increase (Parsons & Morrison, 1978) or decrease (McMillan *et al.*, 1985) in chromosome number and similar DNA content (D'Incalci *et al.*, 1983) have been reported.

The finding that B16V and B16VDXR lines have similar spontaneous metastatic behaviour but different colonization capability extends similar observations of lack of coincidence of the two properties as reported for B16 melanoma (Stackpole, 1981) and other murine tumours (Price *et al.*, 1984). The lower colonization potential of the resistant line is directly related to the cloning efficiency which has been found to be 10 times lower for the resistant line (Supino *et al.*, 1986) and confirm similar findings on B16 melanoma subclones (Stackpole *et al.*, 1985*b*). Such a correlation does not exist between the cloning efficiency and the tumourigenicity of these two lines as already reported for B16 melanoma cells in culture (Kreider & Schomayer, 1975). In addition it should be noted that, at variance with what has been reported for other resistant tumour lines (Biedler *et al.*, 1975; Biedler *et al.*, 1983) only a slight decrease in tumourigenic potential is associated with development of resistance in our line.

The enhancement in metastasis formation not associated with an increase in survival time, sometimes observed in DX treated mice bearing the resistant lines, is an interesting finding with potentially important clinical implications. This unfavourable result might be due to the immunosuppressive effect of chemotherapy on moderately or markedly antigenic tumours (Elbe *et al.*, 1973; Nowak et al., 1973). In fact, it has been reported that treatment with anticancer drugs may lead to induction of new antigens on tumour cells (Nicolin *et al.*, 1972).

The analysis of the relationship between the degree of resistance *in vitro* and the sensitivity to DX treatment *in vivo*, show that the B16VDXR line retained significant responsiveness *in vivo* in spite of a RI value of 69 and this argues about the relationship between *in vitro* and *in vivo* resistance results. From the results obtained in our study this discrepancy might be explained by the latency differences between sensitive and resistant cells with consequent *in vivo* predominance of sensitive cells, with shorter latency, probably still present in the resistant line at that particular RI. This possibility is supported by the fact that if resistant cells were inoculated together with similar percentages of sensitive cells, the resultant tumour behaved as if it was sensitive both in terms of latency and sensitivity.

The loss also of the resistant phenotype during few *in vivo* transplants might be due to latency differences between sensitive and resistant cells and to the procedure used for tumour propagation since each transplant has been performed few days after the tumours had become palpable, i.e. when most likely sensitive cells with shorter latency were replicating.

Our results on DX pharmacokinetics on the resistant line confirm the data obtained *in vitro* on several rodent and human tumour cell lines (Giavazzi *et al.*, 1983; Danø, 1983; Inaba *et al.*, 1979; Howell *et al.*, 1984; Rogan *et al.*, 1984) in which the induction of DX-resistance is associated with decreased DX retention. The lack of activity of DX against the resistant line even if administered more frequently, suggests that decreased drug retention is probably only one component of resistance. In fact, B16VDXR cells have also shown when incubated with DX *in vitro*, a different drug intracellular distribution with lower nucleus/cytoplasm ratio compared to the sensitive cells (Supino *et al.*, 1986).

In conclusion, our results obtained with this cell line, which when first transplanted *in vivo* shows resistance to DX treatment, indicate this model to be suitable for *in vivo* studies of the mechanisms of resistance to DX and for selecting non-cross-resistant drugs and drugs able to circumvent DX-resistance.

The authors wish to thank Dr E. Prosperi (University of Pavia, Italy) for flow cytometry analysis, Mr Roberto Carsana, Miss Loredana Cleris and Mr Marco Busani for excellent technical help and Mrs Grazia Barp for secretarial assistance.

### References

BIEDLER, J.L., RIEHM, H., PETERSON, R.H.F. & SPENGLER, B.A. (1975). Membrane-mediated drug resistance and phenotypic reversion to normal growth behaviour of Chinese hamster cells. *J. Natl Cancer Inst.*, **55**, 671.

BIEDLER, J.L., CHANG, T., MEYERS, M.B., PETERSON, R.H.F. & SPENGLER, B.A. (1983). Drug resistance in Chinese hamster lung and mouse tumor cells. *Cancer Treat. Rep.*, **67**, 859.

DANØ, K. (1972). Development of resistance to adriamycin (NSC-123127) in Ehrlich ascites tumor *in vivo*. *Cancer Chemother. Rep.*, **56**, 321.

DANØ, K. (1983). Active outward transport of daunomycin in resistant Ehrlich ascites tumor cells. *Biochim. Biophys. Acta.*, **323**, 466.

D'INCALCI, M., TORTI, L., DAMIA, G., ERBA, E. & GARATTINI, S. (1983). Ovarian reticular cell sarcoma of the mouse (M5076) made resistant to cyclophosphamide. *Cancer Res.*, **43**, 5674.

ELBE, B., NOWAK, C., ARNOLD, W. & BENDER, E. (1973). Untersuchungen uber den Einfluss einer Vorbehandlung mit Cyclophosphamid, Ribo-Azauracil und Mercaleukin auf die experimentelle Metastasierung. II. Auf Chemisch induzierte Sarkoma von Maus un Ratte. *Arch. Geschwulstforsch.*, **41**, 137.

FORMELLI, F., CARSANA, R. & POLLINI, C. (1986). Comparative pharmacokinetics and metabolism of doxorubicin and 4-demethoxy-4′-0-methyldoxorubicin in tumor bearing mice. *Cancer Chemother. Pharmacol.*, **16**, 15.

GERAN, R.I., GREENBERG, N.H., MACDONALD, M.M., SCHUMACHER, A.M. & ABBOTT, B.J. (1972). Protocols for screening chemical agents and natural products against animal tumors and other biological systems (Third edition). *Cancer Chemother. Rep.*, **3**, 2.

GIAVAZZI. R., SCHOLAR, E. & HART, R. (1983). Isolation and preliminary characterization of an adriamycin-resistant murine fibrosarcoma cell line. *Cancer Res.*, **43**, 2216.

HART, I.R., TALMADGE, J.E. & FIDLER, I.J. (1983). Comparative studies on the quantitative analysis of experimental metastatic capacity. *Cancer Res.*, **43**, 400.

HOWELL, N., BELLI, T.A., ZACZKIEWICZ, L.T. & BELLI, J.A. (1984). High-level unstable adriamycin resistance in a Chinese hamster mutant cell line with double minute chromosomes. *Cancer Res.*, **44**, 4023.

INABA, M., KOBAYASHI, H., SAKURAI, Y. & JOHNSON, R.K. (1979). Active efflux of daunorubicin and adriamycin in sensitive and resistant sublines of P388 leukemia. *Cancer Res.*, **39**, 2200.

KREIDER, J.W. & SCHOMAYER, M.E. (1975). Spontaneous maturation and differentiation of B16 melanoma cells in culture. *J. Natl Cancer Inst.*, **55**, 641.

MACMILLAN, T.J., STEPHENS, T.C. & STEEL, G.G. (1985). Development of drug resistance in a murine mammary tumour. *Br. J. Cancer*, **52**, 823.

MATTERN, J., WAYSS, K. & VOLM, M. (1981). Effect of five antineoplastic agents on tumor xenografts with different growth rates. *J. Natl Cancer Inst.*, **72**, 1335.

NICOLIN, A., VADLAMUDI, S. & GOLDIN, A. (1972). Antigenicity of L1210 leukemic sublines induced by drugs. *Cancer Res.*, **32**, 653.

NOWAK, C., ELBE, B., ARNOLD, W. & BENDER, E. (1973). Untersuchungen uber den Einfluss einer Vorbehandlung mit Cyclophosphamid, Ribo-Azauracil und Mercaleukin auf die experimentelle Metastasierung im syngenen Tumor-Wirt-System. I. Auf ein spontanes Mammakarzinom der Maus. *Arch. Geschwulstforsch.*, **41**, 1.

PARSONS, P.G. & MORRISON, L. (1978). Melphalan-induced chromosome damage in sensitive and resistant human melanoma cell lines. *Int. J. Cancer*, **21**, 438.

PRICE, J.E., CARR, D. & TARIN, D. (1984). Spontaneous and induced metastasis of naturally occurring tumours in mice: analysis of cell shedding into the blood. *J. Natl Cancer Inst.*, **73**, 1319.

RIORDAN, J.R. & LING, V. (1985). Genetic and biochemical characterization of multidrug resistance. *Pharmacol. Ther.*, **28**, 51.

ROGAN, A.M., HAMILTON, T.C., YOUNG, R.C., KLECKER, R.W. Jr. & OZOLS, R.F. (1984). Reversal of adriamycin resistance by verapamil in human ovarian cancer. *Science*, **224**, 994.

SCHABEL, F.M., SKIPPER, H.E., TRADER, M.W., LASTER, W.R. Jr., GRISWOLD, D.P. Jr. & CORBETT, T.H. (1983). Establishment of cross-resistance profiles for new agents. *Cancer Treat. Rep.*, **67**, 905.

SEEBER, S., OSIEKA, R., SCHMIDT, C.G., ACHTERRATH, W. & CROOKE, S. (1982). *In vivo* resistance towards anthracyclines, etoposide, and *cis*-diamminedichloroplatinum (II). *Cancer Res.*, **42**, 4719.

STACKPOLE, C.W. (1981). Distinct lung-colonizing and lung metastatizing cell populations in the B16 mouse melanoma. *Nature*, **289**, 798.

STACKPOLE, C.W., ALTERMAN, A.L. & FORNABAIO, D.M. (1985*a*). Growth characteristics of clonal cell population constituting a B16 melanoma metastasis model system. *Invas. Metast.*, **5**, 125.

STACKPOLE, C.W., FORNABAIO, D.M. & ALTERMAN, A.L. (1985*b*). Phenotypic interconversion of B16 melanoma clonal cell population: relationship between metastasis and tumor growth rate. *Int. J. Cancer*, **35**, 667.

SUPINO, R., PROSPERI, E., FORMELLI, F., MARIANI, M. & PARMIANI, G. (1986). Characterization of a doxorubicin-resistant murine melanoma line: studies on cross-resistance and its circumvention. *Br. J. Cancer*, **54**, 33.